# Civil society’s response to emerging public health events in China

**DOI:** 10.7189/jogh.10.010364

**Published:** 2020-06

**Authors:** Weibin Cheng, Gifty Marley, Huipeng Liao, Weiming Tang

**Affiliations:** 1 Institute for Healthcare Artificial Intelligence Application, Guangdong Second Provincial General Hospital, Guangzhou, China; 2 School of Public Health, Nanjing Medical University, Nanjing, China; 3 Dermatology Hospital of Southern Medical University, Guangzhou, China; 4 Institute of Global Health and STD Research, Southern Medical University, Guangzhou, China; 5 University of North Carolina Project-China, Guangzhou, China

In response to the coronavirus disease 2019 (COVID-19) outbreak, China has been imposed an unprecedented collective effort to control and limit the spread of the virus. While these efforts have been spearheaded by the state, civil societies such as volunteers, social workers, community-based organizations (CBO), and charity foundations and members of the public have contributed essentially in many ways in assisting the control and prevention efforts, supporting medical staff on the frontlines, and aiding the vulnerable groups most seriously impacted by the lockdowns in Hubei and other areas. Civil society refers to space for collective action around shared interests, purposes, and values, generally distinct from government and commercial for-profit actors [1]. This observation is evidential proof that reflects the evolution of civil societies in China.

First, civil societies worked together in promoting personal hygienic, keeping social distance, and delivering medicine and life supplies. According to an online survey (n = 20325) conducted in mainland China [2], 73% to 81% of people wore a mask when walking on the street, which was much higher than during the SARS outbreak in Hongkong, while the improving of the using of the masks are largely due to the propaganda by the civil society on social media. Meanwhile, people curtailed social activities during Chinese Spring Festival by promoting the cancellation of the most important Chinese tradition: “Bainian”, of which relatives and friends visit each other for reunion and greeting a happy new year. These actions are essential for making the government’s social distancing strategy work for controlling of community transmission [3]. Besides, health workers were voluntarily petitioned to support in Hubei province, and around 42000 people health workers are eventually worked there for epidemiological evaluation, quarantine, and treatment. In addition, a group of volunteers initiated an online clinic with hundreds of doctors to provide tele-visits to people asking for medical advice in Hubei, which great relief fear, panic and even deaths in the first two weeks after the city’s lockdown.

**Figure:**
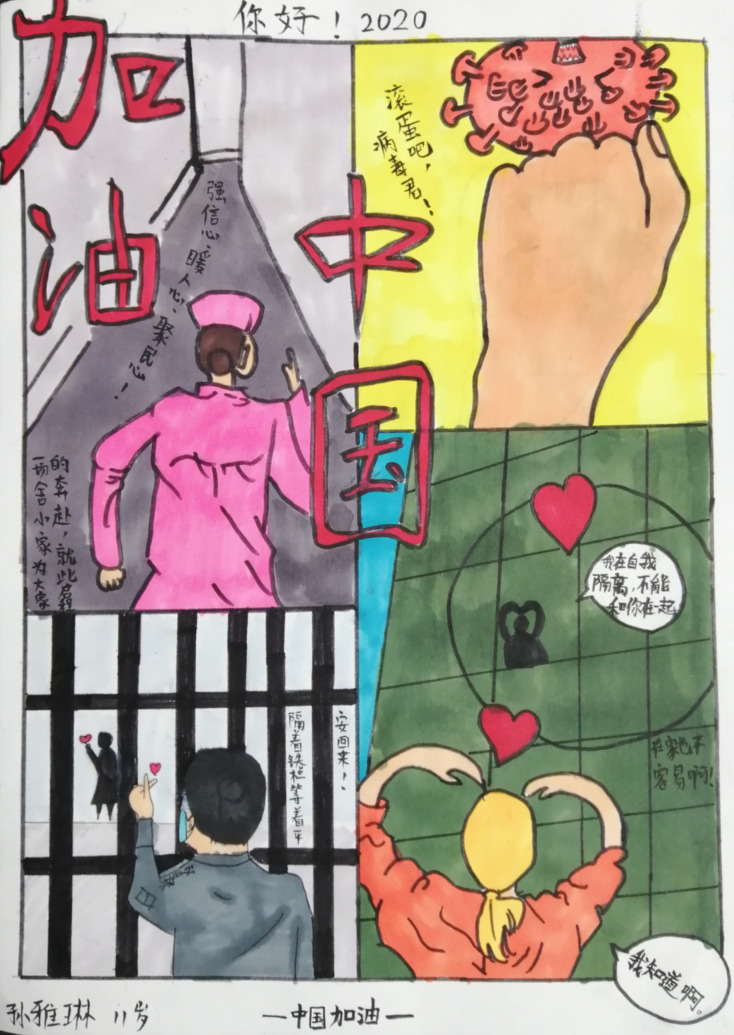
Photo: This is a painting from a boy, drawn to comfort his mother who is a physician had been supporting Hubei during the COVID-19 outbreak. Permission has been granted.

Second, civil societies promoted social campaigns aimed to reduce the stigma towards residences of Hubei provinces in China, solicited funding and medical materials to support health care workers, and advocated for legislation to stop the eating of wild animals. Many ordinary individuals of the public across China have shown a great willingness to donate money and supplies to help the people affected by the epidemic. This should come as no surprise, as it repeats the pattern witnessed after the Wenchuan earthquake and other natural disasters of recent years, when there was a huge wave of donations and volunteering from ordinary citizens [4]. After it was made known that frontline health care providers had run out of personal protective materials, the civil society persistently advocated on different social media platforms and solicited tens of millions of medical supplies, which dramatically relieved the shortage of medical supplies in Hubei province [5]. Meanwhile, thousands of volunteers played a role in maintaining social order and assisting quarantined people to provide daily necessities in different corners of the city. To promote legislation against the consumption of wild animals across the country, nine civil society organizations have jointly made a proposal to the Legal Work Committee of the Standing Committee of the National People’s Congress to promote the amendment of Chinese Wildlife Protection Law.

Furthermore, the Chinese civil societies have actively engaged in post-hoc services to take care of emergency needs unmet by an overwhelmed government [6]. As an important part of the social forces and the professional and technical personnel engaged in specialized social service work, the majority of social workers are actively involved in epidemic prevention and related work. After the lockdown of cities, civil society has continued to find new solutions to assist vulnerable groups whose needs may not be met through official channels. For example, to address the mental health of infected residents as well as that of their families, an online caring group consisting of licensed social workers and psychologists was formed to provide psychosocial support. Statistics shows that, this informal online network provided an average of 200 online consultations daily from February 8 to 13, after which the government-initiated psychological counselling services started to kick in, ensuring that more affected residents could receive psychological support care [7,8]. This facilitated the development of the Chinese public contingency plan and improved the post-hoc mental health service for both patients and general populations. Meanwhile, the grassroot community also contributed in constructive and creative ways to provide aid for Hubei and assist the most vulnerable groups in a time of crisis. Due to the lockdown of Wuhan city or quarantine in the communities, people living with HIV/AIDS (PLHA) were faced with running out of antiretroviral drugs and risk of HIV infection status disclosure if they turned to the local community health staff for help [9]. A CBO which serves for LGBT community voluntarily to aid claiming and delivering ARV drugs from the designated hospital, which has aided over 1500 PLHAs continuing their antiretroviral treatment [10].

As of response to the outbreak of COVID-19, several lessons can be drawn from the civil society’s participation. First, timely and transparent disclosure of the outbreak information and public communication is a prerequisite for civil society participation. Second, the civil society can help the effective implementation of epidemic control and prevention measures which needs unmet by an overwhelmed government. Third, to curb the outbreak of infectious disease should be the responsibility of every citizen. However, the overall effectiveness of the civil society participation in aiding to prevent and control of infectious diseases outbreak needs to be assessed in future empirical studies.

A thousand miles begins with a single step. Engagement of civil society in response to the COVID-19 outbreak indicated the growth of Chinese civil society. However, as its development is still at a very early stage, strategies and policies that aim to create an opening environment for its further growth are needed. In addition, civil societies in China should learn from the experiences of other countries, and increasingly improve their ability in response to emergency public health events.
